# Structural insights into SOD1: from in silico and molecular dynamics to experimental analyses of ALS-associated E49K and R115G mutants

**DOI:** 10.3389/fmolb.2025.1532375

**Published:** 2025-02-25

**Authors:** Seyed Mahdi Hosseini Faradonbeh, Bagher Seyedalipour, Nasrin Keivan Behjou, Kimiya Rezaei, Payam Baziyar, Saman Hosseinkhani

**Affiliations:** ^1^ Department of Molecular and Cell Biology, Faculty of Basic Science, University of Mazandaran, Babolsar, Iran; ^2^ Department of Biochemistry, Faculty of Biological Sciences, Tarbiat Modares University, Tehran, Iran

**Keywords:** protein structure, protein stability, human superoxide dismutase-1, E49K, R115G, protein aggregation, ALS

## Abstract

Protein stability is a crucial characteristic that influences both protein activity and structure and plays a significant role in several diseases. Cu/Zn superoxide dismutase 1 (SOD1) mutations serve as a model for elucidating the destabilizing effects on protein folding and misfolding linked to the lethal neurological disease, amyotrophic lateral sclerosis (ALS). In the present study, we have examined the structure and dynamics of the SOD1 protein upon two ALS-associated point mutations at the surface (namely, E49K and R115G), which are located in metal-binding loop IV and Greek key loop VI, respectively. Our analysis was performed through multiple algorithms on the structural characterization of the hSOD1 protein using computational predictions, molecular dynamics (MD) simulations, and experimental studies to understand the effects of amino acid substitutions. Predictive results of computational analysis predicted the deleterious and destabilizing effect of mutants on hSOD1 function and stability. MD outcomes also indicate that the mutations result in structural destabilization by affecting the increased content of β-sheet structures and loss of hydrogen bonds. Moreover, comparative intrinsic and extrinsic fluorescence results of WT-hSOD1 and mutants indicated structural alterations and increased hydrophobic surface pockets, respectively. As well, the existence of β-sheet-dominated structures was observed under amyloidogenic conditions using FTIR spectroscopy. Overall, our findings suggest that mutations in the metal-binding loop IV and Greek key loop VI lead to significant structural and conformational changes that could affect the structure and stability of the hSOD1 molecule, resulting in the formation of toxic intermediate species that cause ALS.

## 1 Introduction

**FIGURE 1:**
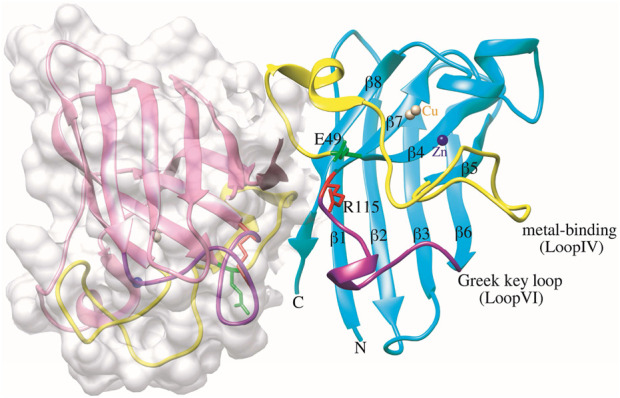
Structure of dimeric human SOD1 in non-pathogen conformation (PDB ID: 2C9V). Chain F is depicted in surface representation for clarity, and in chain A, E49 and R115 residues are shown green and red, respectively. The metal-binding loop IV (residues 49–82) and the Greek key loop VI (residues 102–115) are functionally significant structural elements of the SOD1 structure, represented in yellow and purple, respectively. The Cu and Zn ions are shown as brown and blue spheres, respectively. The graphic was generated using UCSF Chimera software.

The stability of a protein is a vital feature that affects protein activity, structure, and regulation. It plays an essential role in evolution, industrial applications, and many diseases (Chen et al., 2020). Protein folding and stability are maintained by interactions among inner and outer residues. The hydrophobic effect, hydrogen bonding, interactions between the tightly packed buried residues, protein electrostatics (Vascon et al., 2020), and flexibility contribute to the stability of a protein (Kamerzell and Middaugh, 2008; Teilum et al., 2011). Studying the biophysical and biochemical principles behind protein stability through theoretical and experimental approaches has become an increasing interest over the past several decades (Kumar et al., 2017; Jahan and Nayeem, 2020; Pereira et al., 2021). Human superoxide dismutase 1 (hSOD1) is an incredibly stable metalloenzyme and has become a paradigm for investigating the destabilizing effect on protein folding and misfolding linked to the fatal neurodegenerative disease, amyotrophic lateral sclerosis (ALS) (Valentine et al., 2005; Rakhit and Chakrabartty, 2006). ALS is known as Lou Gehrig’s and motor neuron disease that causes progressive, lethal, degenerative disorder of motor neurons, and the disease kills its victim typically 3–5 years after initial diagnosis (Taylor et al., 2016). About 20% of FALS patients are linked to mutations in the SOD1 gene (Rosen et al., 1993). Mature and functional SOD1 is a 32 kDa homodimeric protein with 153 amino acid residues in each subunit that catalyzes the conversion of the toxic superoxide anion to hydrogen peroxide and molecular oxygen. Each monomer is composed of an eight-stranded antiparallel β-barrel connected by three external loops (Strange et al., 2003). Furthermore, each monomeric subunit has two metal-binding sites, one for copper and one for zinc, and an intramolecular disulfide bond ranging from Cys57 to Cys146 stabilizes them (Baziyar et al., 2022). The two active site loops are metal-binding loop IV (residues 49–82) and electrostatic loop VII (residues 121–142). Loop IV is divided into three subregions: the dimer interface region (residues 49–54), the disulfide bond region (residues 55–61), and the zinc-binding region (residues 62–82) (Hwang et al., 2010). Finally, a third and shorter loop, referred to as the Greek key loop VI (residues 102–115), forms a plug at one pole of the β-barrel and enhances dimer interface stability (Trist et al., 2021). Several residues, including V5, V7, K9, I17, E49-T54, I113-R115, and V148-Q153, are involved in the intra-subunit interactions at the dimer interface of SOD1 (Ghosh et al., 2020). Some novel mutations related to FALS were identified by Boukaftan et al. in exon 2 of the hSOD1 gene. A new mutation causes glutamic acid, a negatively charged amino acid, to be replaced by lysine, a positively charged amino acid (E49K), while the amino acid Glu 49 is not generally conserved across species (Boukaftane et al., 1998). Kostrzewa et al. reported a new mutation in exon 4 of the hSOD1 gene, which causes arginine to be replaced by glycine (R115G). This substitution constitutes a significant amino acid change from a positively charged (Arg) to a neutral (Gly) amino acid, while amino acid Arginine 115 is highly conserved in different species, which indicates an important function of Arg at this position of the enzyme. The location of Arg 115 in the dimer contact site of the SOD1 protein suggests its relevance to the structural integrity of the enzyme (Kostrzewa et al., 1994). Up to now, more than 200 mutations have been reported in FALS patients (http://alsod.iop.kcl.ac.uk/) (Abel et al., 2012; Basith et al., 2022; Suzuki et al., 2023). In the last few years, significant efforts have been implicated to reveal the effects of ALS-associated mutations on the biochemical and biophysical properties of hSOD1 (Namadyan et al., 2023; Zaji et al., 2023). Remarkably, the location of these mutations is all over the SOD1 structure, which makes it challenging to determine the exact destabilizing process (Tompa et al., 2020). These mutations lead to reduced metal ion content, decreased net charge (in some cases), disruption of the surface hydrogen bond network, increase in the hydrophobicity, changes in the flexibility, and alternations in the functional characteristics of SOD1, therefore play a crucial role in the formation of toxic aggregates and the pathology of ALS (Antonyuk et al., 2005; Khare and Dokholyan, 2006; Byström et al., 2010; Tompa and Kadhirvel, 2020; Mavadat et al., 2023a; Mavadat et al., 2023b). Against this background, the current study was an attempt to shed light on the structural and dynamical features of the holo forms of the wild-type hSOD1 (WT-hSOD1) (Figure 1), E49K, and R115G mutants in metal-binding loop IV and Greek key loop VI, respectively, using computational predictions, molecular dynamics (MD) simulations, and experimental studies to characterize some physicochemical properties and their relation to enzymatic activity, stability, and protein aggregation on an atomic and a molecular level.

## 2 Materials and methods

### 2.1 Computational methods

#### 2.1.1 Sequence analysis and dataset

The amino acid sequence of hSOD1 was achieved from the UniProt database (ID: P00441) (Consortium, 2015). The structure for the WT-SOD1 protein was acquired through the Protein Data Bank [PDB ID: 2C9V] (Berman et al., 2000). The PDB ID: 2C9V structure was employed because it has the highest atomic resolution (1.07Å) compared to other SOD1 structures (Pereira et al., 2021).

#### 2.1.2 Functional and stability prediction analysis

The functional effects of E49K and R115G mutants on hSOD1 protein were predicted using the following algorithms: Panther (Mi et al., 2005), PolyPhen-1 (Ramensky et al., 2002), PolyPhen-2 (Adzhubei et al., 2013), MAPP (Stone and Sidow, 2005), Phd Snp (Capriotti et al., 2006), SIFT (Ng and Henikoff, 2003), SNAP (Bromberg and Rost, 2007), and PredictSNP for accurate predictions along with the prediction score. The majority of these tools are designed to predict whether a certain replacement is neutral or deleterious by using a variety of factors obtained from the evolutionary, physicochemical, or structural characteristics (Bendl et al., 2014). In addition, the effects of mutants on hSOD1 stability were analyzed using the different prediction web servers, viz., DUET (Pires et al., 2014a), I-Mutant2.0 (Capriotti et al., 2005), mCSM (Pires et al., 2014b), DDGun (Montanucci et al., 2019), SAAFEQ-SEQ (Li et al., 2021), DynaMut2 (Rodrigues et al., 2021), and i-Stable. These algorithms evaluated the effect of mutation on hSOD1 stability by using free energy change (ΔΔG) calculations, which could report positive and negative values for stabilizing and destabilizing mutations, respectively (Chen et al., 2013).

#### 2.1.3 Prediction of the effect of mutations on protein structure and conformation

Using Chimera software (Pettersen et al., 2004) version 1.16 (PDB ID: 2C9V), we have observed changes in the interactions of neighboring amino acids and structural alterations in E49K and R115G mutants compared to WT-hSOD1. The software was used to scan the three-dimensional structure of SOD1 proteins and then replace the native amino acid with the candidate to demonstrate the impact.

#### 2.1.4 Prediction of the effect of mutations on the molecular net charge

The molecular net charge of WT-hSOD1, E49K, and R115G mutants was computed at pH 7.4 using the Prot pi Protein Tool (https://www.protpi.ch/Calculator/ProteinTool). In addition, using Chimera software (PDB ID: 2C9V), we have visualized the electrostatic potential map onto the molecular surface of WT-hSOD1 and E49K and R115G mutants.

#### 2.1.5 Molecular dynamics simulations

Molecular Dynamics (MD) simulations of the WT-hSOD1 protein and its mutants (E49K and R115G) were performed using the GROMACS 2022.6 software package (Van Der Spoel et al., 2005). The program Visual Molecular Dynamics 1.9.3 was utilized to induce the mutations (E49K and R115G) on the crystallographic structure of the WT-hSOD1 dimer (PDB ID: 2C9V). The force field CHARMM36-JUL2022 was selected for the simulations. The molecules were solvated in a triclinic box with TIP3P water molecules, and to maintain overall charge neutrality, sodium (Na⁺) and chloride (Cl⁻) ions were introduced to substitute some solvent molecules. The simulations were performed in four distinct stages. In the initial stage, the energy in the system was minimized using the steepest descent algorithm to eliminate additional forces acting on the atoms, thereby relaxing the systems. The subsequent second and third stages maintained a temperature of 300 K and a pressure of 1 bar, achieved through two 1-nanosecond (ns) simulations conducted in the canonical (NVT) and isothermal-isobaric (NPT) ensembles, with position restraints applied to the heavy atoms. The final phase removed position restraints on all atoms and included a 300 ns simulation with a time step of 2 femtoseconds (fs), allowing for trajectory recording to extract various physical properties. Copper (Cu) and zinc (Zn) ions were restrained to their designated binding sites throughout the simulations. Subsequently, the MD trajectories were analyzed using the following GROMACS package distribution programs: gmx rms, gmx hbond, gmx gyrate, gmx sasa, gmx rmsf, and gmx dssp to compute root-mean-square deviation (RMSD), hydrogen bonds (H-bond), radius of gyration (Rg), solvent-accessible surface area (SASA), root-mean-square fluctuation (RMSF), and secondary structure (SS), respectively. PCA is referred to as essential dynamics (ED) when used for the analysis of molecular dynamics (MD) simulations (Pereira et al., 2020). Theoretical aspects of PCA have been thoroughly discussed in prior studies (Zaji et al., 2023). The GROMACS tools performed PCA of the WT-SOD1, E49K, and R115G mutants in each system. Additionally, the popular conformations were examined utilizing the clustering scheme of GROMACS with a cutoff of 0.2 nm.

#### 2.1.6 Free energy landscape

The free energy landscape of a protein was obtained using the conformational sampling approach, which produces the near-native structural conformation. The free energy landscape (FEL) for conformational sampling of the WT-SOD1, E49K, and R115G mutants was constructed using simulated trajectory data. Consequently, FEL was derived by mixing RMSD and Rg trajectories with free energy according to the following Equation 1:

$$\Delta G\;\left(p1,p2\right)=-k_{B}T\ln\rho \left(p1,p\;2\right)$$ (1)

Where in, ΔG depicts the Gibbs free energy of state, *k*_B_ represents the Boltzmann constant and *T* corresponds to the temperature of the simulation. The FEL’s reaction coordinates *p*1 and *p*2, are shown by joint probability distribution *ρ* (*p*1, *p*2) (Papaleo et al., 2009; Noorbakhsh Varnosfaderani et al., 2023).

#### 2.1.7 Binding free energy and per-residue free energy decomposition analysis

The g_mmpbsa tool was employed to calculate the binding free energy of the WT-hSOD1 and its mutants (E49K and R115G). g_mmpbsa computed the binding free energy of the complex structure using the Molecular Mechanics Poisson–Boltzmann Surface Area (MM/PBSA) methodology (Basith et al., 2022). The following Equation 2 is used to determine the binding free energy ΔG_binding_ of a complex:

$$\Delta G_{\text{binding}}=\Delta G_{\text{complex}}\;–\;\left(\Delta G_{\text{protein}1}+\Delta G_{\text{protein}2}\right)$$ (2)

Here, Δ_Gcomplex_ demonstrates the total MMPBSA energy of the protein-protein complex; ΔG_protein1_ and ΔG_protein2_ are solution-free energies of the individual protein_1_ and protein_2_, respectively. The free energy of the individual existence can be expressed as follows Equation 3:

$$\Delta G=E_{\text{MM}}+G_{\text{solvation}}-\text{TS}$$ (3)

E_MM_ shows the average molecular mechanical potential energy in the vacuum; G_solvation_ defines the free energy of solvation. T and S explain the temperature and entropy, respectively, and together TS exhibits the entropic contribution to the free energy in vacuum. In addition, the E_MM_ contains both bonded and nonbonded interactions of the molecules, including the bond angle, torsion, and electrostatic (E_elec_) and van der Waals (E_vdw_) interactions. Last, the free energy of solvation and G_solvation_ include both electrostatic and nonelectrostatic (G_polar_ and G_nonpolar_) components (Kumari et al., 2014). To gain more insights into the residue contributions, the residues of SOD1 responsible for the interface surface were computed using the MM-PBSA per residue free energy decomposition method (mm_pbsa module) of GROMACS-CHARMM36. These calculations were based on the same snapshots as those used in the binding free energy calculations.

### 2.2 Experimental methods

#### 2.2.1 Materials

A plasmid extraction kit was purchased from GeneAll (South Korea). *Pfu* polymerase and Ni-NTA agarose resin were obtained from Agarose Bead Technologies (ABT) and Wizbiosolutions, respectively. *Dpn I* was purchased from Thermo Scientific. Phenylmethylsulfonyl fluoride (PMSF), Kanamycin, Dialysis tubing cellulose membrane (12,400 Da cutoff), 1-Anilinonaphthalene-8-sulfonate (ANS), and Isopropyl-D-thiogalactopyranoside (IPTG) were acquired from Sigma. Dithiothreitol (DTT), Yeast Extract, Tryptone, and Agarose were obtained from Bio Basic. Copper sulfate (CuSO_4_) and zinc sulfate (ZnSO_4_) were purchased from Merck. All other reagents were of analytical reagent grade and were used without further purification.

#### 2.2.2 Site-directed mutagenesis and WT-hSOD1 and mutant expression and purification

The recombinant pET28a-hSOD1 served as the template to create E49K and R115G mutations using *Pfu* DNA polymerase and the QuickChange site-directed mutagenesis method. Two mutagenesis primers for E49K were designed: forward primer (5′TGGATTCCATGTTCAT**AAG**TTTGGAGATA ATACAGCAG-3′) with mutation site (highlighted) and reverse primer (5′-AGCCTGCTGTATTAT CTCCAAA**CTT**ATGAACATGG-3′) with mutation site (highlighted). Additionally, two primers were designed for the R115G mutation: the forward primer (5′-ACCATTGCATCATTGGC**GGC**A CACTGGTGG-3′) with mutation site (highlighted) and the reverse primer (5′-TCATGGACCACCA GTGT**GCC**GCCAATGATGC-3′) with mutation site (highlighted) were designed to amplify pET28a-hSOD1 as a template. The product PCR is digested with *Dpn I* to destroy methylated parental plasmid DNA. The recombinant expression plasmid was transformed into *E. coli* BL21 (DE3) competent cells for protein production by heat shock. The protein was expressed, purified using a Ni-NTA affinity column, and characterized. The purified SOD1 proteins were reconstituted with CuSO_4_ and ZnSO_4_ by dialysis in 3 main steps: metal removal, metal charging, and removal of unbound metals at 4°C, as previously described (Mohammadi et al., 2024). The concentration of purified WT-hSOD1, E49K, and R115G mutant proteins was determined by the Bradford method. Using a 12.5% SDS-PAGE electrophoresis, a single band of about 16 kDa was observed with high purity (data not shown).

#### 2.2.3 Enzymatic activity assay of WT-hSOD1, E49K and R115G mutants

Enzyme activity assay was performed in a volume of 300 μL composed of Tris-HCl buffer (50 mM) containing EDTA (1 mM; pH 8.2), 0.1 mM pyrogallol (dissolved in 10 mM HCl), and 20 μL of enzyme purified by the Marklund method (Marklund and Marklund, 1974) in a microplate spectrophotometer reader (Epoch, BioTek, USA). The rate of pyrogallol autoxidation depends on the availability of superoxide and is monitored spectrophotometrically (at 420 nm, 5 min, 30 s intervals) by a color change. One unit of SOD1 activity is defined as the enzyme amount inhibiting pyrogallol autoxidation by 50% per minute, with specific activity measured in U/mg protein (Tiwari et al., 2019).

#### 2.2.4 Spectroscopic characterization

Intrinsic (Trp) fluorescence in proteins is a widely used tool for monitoring protein changes, providing detailed information on dynamics and conformational changes (Vivian and Callis, 2001; Ghisaidoobe and Chung, 2014). Intrinsic fluorescence measurements were carried out using a spectrofluorometer JASCO fluorescence spectrophotometer (FP-8300) with excitation fixed at 295 nm, and emission spectra were measured between 300 and 500 nm (Exc/Em slit widths = 5/10 nm, respectively). Extrinsic fluorescence studies using ANS are applied to monitor exposed hydrophobic pockets (Hawe et al., 2008). ANS fluorescence emission spectra were measured from 400 to 700 nm, with excited at 370 nm. The purified enzyme concentration was 20 μg/mL for each assay in phosphate buffer (20 mM, pH 7.4). Additionally, intrinsic fluorescence and extrinsic fluorescence emission spectra were investigated under amyloidogenic conditions (DTT 50 mM, Tris-HCl 50 mM, and KSCN 0.16 M). The concentration of ANS was 30 μM with a protein-to-ANS ratio of 1:30.

#### 2.2.5 Fourier-transform infrared spectroscopy

Using potassium bromide (KBr) tablets, FTIR spectroscopy (BRUKER TENSOR 27, Germany) was used to examine the protein’s secondary structure of the WT-SOD1, E49K, and R115G mutants. The infrared spectra were recorded at a resolution of 4 cm^−1^, spanning the range of 400–4,000 cm^−1^. The protein samples were investigated under amyloidogenic conditions (50 mM Tris-HCl, 50 mM DTT, 0.16 M KSCN) at a concentration of 25 μM. Subsequently, 10 μL of each sample was used for FTIR evaluations. The buffer baseline was then subtracted before taking each spectrum (Chowdhury et al., 2019). The peak assignments were accordingly made by the previously described spectral components associated with various secondary structure elements (Gregoire et al., 2012). The raw spectra in the amide I region (1,600–1,700 cm^−1^) were further processed, using the OriginPro 2021 software package. In the FTIR curve fitting method for analyzing the amide I band components, Fourier self-dissociation (FSD) and second derivative spectra were used to elucidate the overlapping peaks in the amide I region.

#### 2.2.6 Characterization of hSOD1 aggregation by ThT fluorescence

The fibrillation kinetics was investigated by thioflavin-T (ThT) fluorescence assay based on the rise in the fluorescence quantum efficiency of ThT after binding to the amyloid fibers. The aggregated SOD1 protein samples, under the conditions inducing amyloid aggregate formation (viz., 0.16 M KSCN, 50 mM DTT, 50 mM Tris-HCl) at 37°C (pH 7.4) and 190 rpm were then incubated. The concentration of the SOD1 variants fibrils (30 μM dimer, 20 mM phosphate buffer, pH 7.4) was also obtained. ThT (20 μM) was then added to the incubated samples at different time intervals. Afterward, fluorescence was recorded with the excitation and emission at 444 nm and 485 nm, respectively. The excitation and emission slit widths were subsequently set at 5 and 10 nm, in that order. The reported data were thus the mean measurements in three replicates. As previously reported (Esmaeili et al., 2020), the fibrillation kinetic parameters were acquired using the following Equation 4:

$$F=\frac{Fmax}{\;1+\exp\left\{-\left[\left(t-tm\right)/\tau \right]\right.}$$ (4)

where *F* and *F*_max_ represent the fluorescence intensity at time *t* and the end time of ThT signal recording, respectively. As well, *t* shows the characteristic time constant and *t*_*m*_ stands for the time through which the half of the amyloid aggregates are formed. *F*_max_ and *τ* are the floating parameters, according to the Best-Fit (BF) method. Furthermore, *k*_*app*_ denotes the apparent rate constant, given by 1/*τ* for the increase in the length of fibrils, and the formula of *t*_*D*_ = *t*_*m*_ - 2*τ* is then practiced to calculate the delay time (*t*_*D*_).

## 3 Results and discussion

### 3.1 Computational section

#### 3.1.1 Functional and stability prediction analysis

The analysis of single nucleotide variations and their prioritization for experimental characterization heavily relies on computational methods to predict the effects of mutations on protein function (Bendl et al., 2014). The effect of mutations on the hSOD1 protein function was analyzed by eight algorithms: PANTHER, SIFT, SNAP, PhD-SNP, MAPP, PolyPhen-1, PolyPhen-2, and Predict-SNP were combined into a consensus classifier, PredictSNP. The results from all those tools predicted that the mutations are deleterious except Polyphen-2 for the E49K mutant, indicating the neutral effect of the mutation on hSOD1 function with a confidence score of 0.68. Using Predict-SNP, the confidence score predicted for E49K and R115G mutants was found to be 0.72 and 0.87, respectively, which suggested that the mutations have a deleterious effect on hSOD1 protein function. The confidence score that was calculated from each tool was tabulated in Table 1. These results demonstrate the importance of using different algorithms to ensure optimal reliability. Protein stability is crucial in thermodynamics, determined by the change in free energy between the folded and unfolded states (Srinivasan and Rajasekaran, 2018). The stability hSOD1 concerning mutations was examined through ΔΔG calculations using several predictive tools available on the web, including i-Stable, I-mutant2.0, DUET, mCSM, DDGun, DynaMut2, and SAAFEC-SEQ. The results of integrated servers computed in this line for E49K and R115G mutations as presented in Table 2 showed destabilizing effects of mutation on the hSOD1 structure. Previous reports have established the role of some mutations on the function and stability of SOD1 using various computational tools (Srinivasan and Rajasekaran, 2017; 2018; Da Silva et al., 2019; Srinivasan and Rajasekaran, 2019).

**TABLE 1:** Functional predictions and confidence scores for the hSOD1 mutants (E49K and R115G) were computed from the top eight algorithms of the PREDICT-SNP server.

Programs	E49K*	R115G**	Prediction
	Confidence score		
Predict SNP	0.72	0.87	Deleterious
MAPP	0.43	0.62	Deleterious
Phd-SNP	0.88	0.88	Deleterious
PolyPhen-1	0.59	0.59	Deleterious
Polyphen-2	0.68	0.81	Neutral*/Deleterious**
SIFT	0.53	0.53	Deleterious
SNAP	0.72	0.81	Deleterious
PANTHER	0.69	0.84	Deleterious

Note: The SNP, prediction was performed at pH = 7.4°C and 25°C. Abbreviations: SNP, single-nucleotide polymorphism.

**TABLE 2:** Stability prediction results using *in silico* analysis of hSOD1 mutants (E49K and R115G).

Programs	E49K	R115G	Results
	∆∆*G* (Kcal/mol)		
i-Stable	−0.244	−0.164	Decreases
AUTO–MUTE SVM	−1.15	−1.25	Decreases
AUTO–MUTE RF	−1.85	−0.22	Decreases
I-mutant2.0 PDB	−0.96	−1.60	Decreases
I-mutant2.0 SEQ	−0.54	−0.89	Decreases
DUET	−1.04	−2.133	Destabilizing
mCSM	−1.268	−2.401	Destabilizing
DDGun	−1	−2.7	Destabilizing
DDGun3D	−0.5	−2.1	Destabilizing
DynaMut2	−0.96	−2.78	Destabilizing
SAAFEC-SEQ	−0.38	−0.64	Destabilizing

Note: In silico analysis for stability, prediction was performed at pH = 7.4°C and 25°C.

#### 3.1.2 Molecular modeling

**FIGURE 2:**
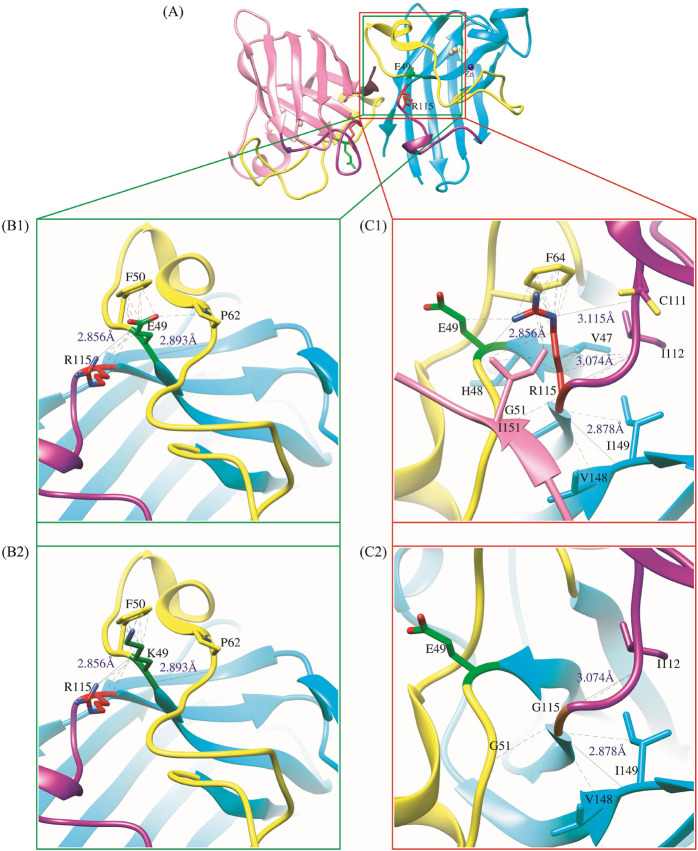
**(A)** A general schematic of WT-hSOD1 and the location of the investigated mutations (E49K and R115G) (PDB ID: 2C9V). **(B1)** Schematic illustrations of the interaction between the Glu49 side chain in WT-hSOD1 and **(B2)** Lys49 side-chain mutant protein (E49K) with the neighboring residues. **(C1)** Schematic demonstrations of the interaction between the Arg115 side chain in WT-hSOD1 and **(C2)** Gly115 side-chain mutant protein (R115G) with the neighboring residues. Hydrogen bonds (distance in Å) are illustrated as a solid line, while close contacts and clashes among residues are represented by a dashed line. The graphic was generated using UCSF Chimera software.

To elucidate the effects of amino acid substitutions, we utilized the UCSF Chimera software for evaluating the impacts of single-point mutations on conformational alterations of the E49K and R115G mutants compared to WT-hSOD1 (Figure 2A), thereby enhancing our understanding of their contribution to disease development. As shown in Figure 2B1, the glutamic acid residue at position 49 (E49) is vital for enabling significant interactions inside protein structures by forming hydrogen bonds and van der Waals interactions. Before the E49K mutation, E49 establishes significant hydrogen bonds with the residues Arg115 and Pro62. These hydrogen bonds promote the three-dimensional structure of the protein to be stabilized and enable important chemical interactions required for appropriate biological function. Apart from its hydrogen bonding features, E49 interacts by van der Waals with Pro62. This connection increases the stability of the protein structure by supporting the integrity of the protein’s core and enhancing the hydrophobic environment. Substituting Lys with Glu at position 49 destabilizes the surrounding area. The absence of van der Waals interaction with Pro62 may modify the spatial arrangement of this residue inside the protein, despite the hydrogen bonds between Arg115 and Pro62 being intact, the alteration in the spatial arrangement of this residue may affect the protein’s overall stability and functionality (Figure 2B2). As illustrated in Figure 2C1, the original residue R115 is crucial for the structural stability and functional interaction of proteins since its various bonds can endow the protein’s integrity. The ability of R115 to form hydrogen bonds with key residues (Glu49, Cys111, Ilu112, Ilu149) of the protein is conspicuous. Besides hydrogen bonding, R115 establishes van der Waals interactions with Phe64 and His48, along with hydrophobic interaction with Val47. Moreover, Arg115 forms a van der Waals interaction with Ilu151 in chain F. These types of interactions contribute significantly to the overall stability of the protein structure. The R115G mutation significantly alters the hydrogen bonding networks and interaction profiles by substituting R115 with G. After this mutation, the hydrogen bonds that were engaged in Glu49 and Cys111 are eliminated, resulting in the local structure of the protein being disturbed. Specifically, Glu49 located in the E49-T54 loop region is crucial for the robust binding of monomers at the dimer interface; any abnormal fluctuations in this loop region might adversely affect the stability of the SOD1 dimer (Ghosh et al., 2020). Notably, Cys111, located on the surface adjacent to the dimer interface of the SOD1 subunit, is oxidized by redox substrates in human wild-type SOD1. These oxidative changes of Cys111 may alter the structure of the SOD1 dimer, leading to monomerization and subsequent aggregation formation of the SOD1 protein (Nagano et al., 2015). Furthermore, the R115G mutation reduces the van der Waals interactions with Phe64, His48, and Ilu151 (chain F) and the hydrophobic contact with Val47, which were formerly sustained by R115. In addition to disrupting these crucial connections that enhance the protein’s dimer stability, the absence of R115 may also reveal hydrophobic pockets typically buried within the protein’s core (Figure 2C2). This exposure may result in increased aggregation tendency.

#### 3.1.3 Molecular net charge analysis

**FIGURE 3:**
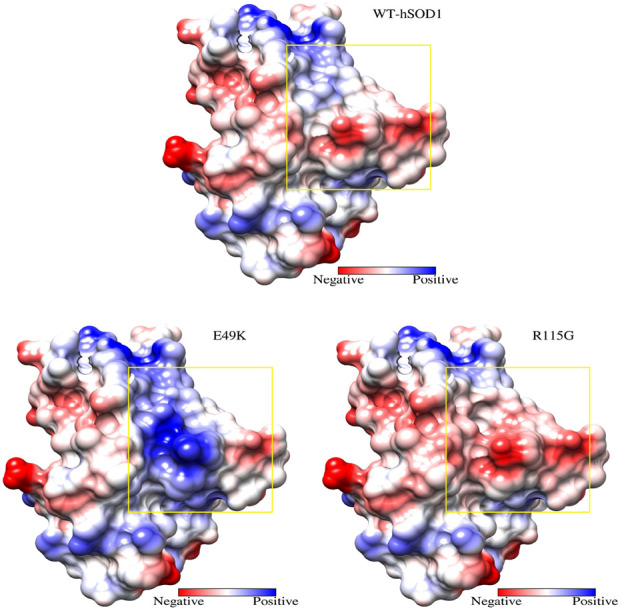
Effects of single-point mutations in WT-hSOD1 (PDB ID: 2C9V) on electrostatic potential. Electrostatic potential was mapped onto the surfaces of WT-hSOD1 (Up) vs. E49K (Reduce net negative charge) and R115G (Increase net negative charge) mutants (Down). Electropositively and electronegatively charged areas are colored in blue and red, respectively. Neutral residues are in white. The graphic was generated using UCSF Chimera software.

The charged and polar side chains of SOD1, similar to other soluble proteins, are located on the protein surface, protrude freely into the solvent, or participate in solvent-accessible salt bridges and hydrogen bonding (Byström et al., 2010). The active site is positively charged and accounts for around 11% of the exposed surface, whereas the remainder is negatively charged (Rakhit and Chakrabartty, 2006). A protein’s net charge is a key physical characteristic that affects its solubility and aggregation (Gitlin et al., 2006). Biomolecules often possess a net negative charge to ensure solubility inside the cellular interior. Each SOD1 monomer normally has a net charge of −6 (Sandelin et al., 2007). Point mutations in SOD1 that cause ALS often reduce the net negative of SOD1 (Prudencio et al., 2009). At pH 7.4 using the Prot pi Protein Tool, the WT-hSOD1, E49K, and R115G mutants have the molecular net charge (−6.52), (−4.53), and (−7.52), respectively. The results obtained from this tool demonstrated a significant alteration in the molecular net charge of both mutants. Notably, for mutant E49K, there was a reduction in net negative charge compared to the wild-type protein. As illustrated in Figure 3, the electrostatic potential was mapped onto the surfaces of WT-hSOD1. Glu49 is located on the outer surface of metal-binding loop IV, surrounded by positively charged His48 and Arg115, which likely serves to neutralize these positive charges and maintain the surface electrostatic potential of SOD1. Mutation of Glu49 to a positively charged residue (Lys) was found to alter the net negative charge (reduce net negative charge) on the protein’s surface (Figure 3), which could reduce the stability of the SOD1 dimer, promote aberrant interactions and misfolding, and/or nucleate aggregation. Arg115 is located on the outer surface of Greek key loop VI, surrounded by negative charge residue (Glu49). On the other hand, an ALS-associated mutation of Arg115 to a neutral residue (Gly) was found to increase the net repulsive charge of the SOD1 (Figure 3). A previous study suggested that two ALS-associated point mutations at the surface residue Glu100 (E100G and E100K) in SOD1, which decrease the net negative charge of the protein by eliminating negatively charged or introducing positively charged amino acids, respectively, could reduce the stability of SOD1 and promote aggregation (Tompa and Kadhirvel, 2020).

#### 3.1.4 MD-based simulation analysis

**FIGURE 4:**
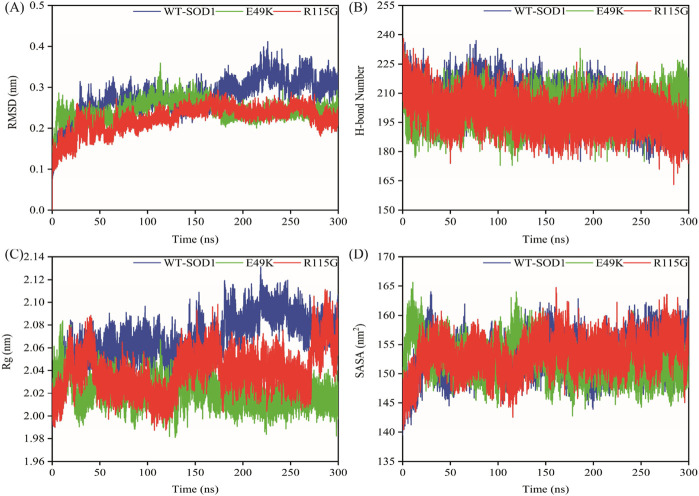
The overall structural changes in WT-SOD1, E49K, and R115G mutants during molecular dynamics simulation. **(A)** RMSD, **(B)** H-bond, **(C)** Rg, and **(D)** SASA plots of WT-SOD1 (blue line), E49K (green line), and R115G mutants (red line).

**FIGURE 5:**
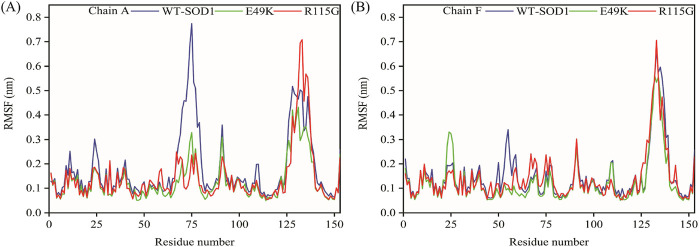
Illustrates the RMSF of the residues of WT-SOD1, E49K, and R115G mutants. **(A)** RMSF (Chain A), **(B)** RMSF (Chain F) plots of WT-SOD1 (blue line), E49K (green line), and R115G mutants (red line).

**FIGURE 6:**
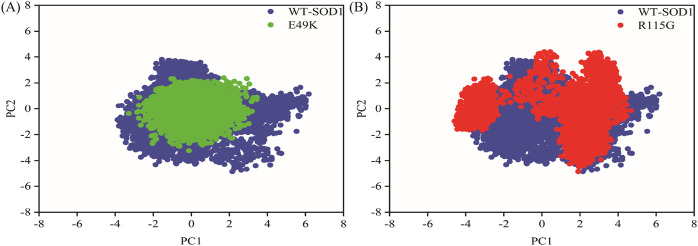
PCA for the WT-SOD1, E49K, and R115G mutants. The first two principal components obtained from the MD trajectories were represented as projections. **(A)** Comparison between the PCA projections for the WT-SOD1 (blue) and E49K mutant (green). **(B)** Comparison between the PCA projections for the WT-SOD1 (blue) and R115G mutant (red).

**FIGURE 7:**
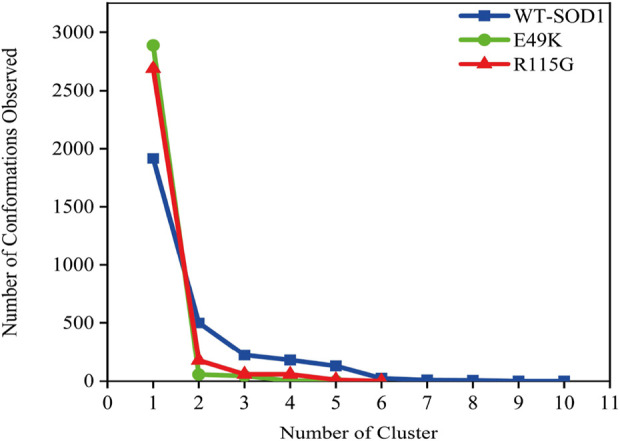
The number of clusters and observed conformations for WT-SOD1 (blue line), E49K (green line), and R115G mutants (red line) by clustering method.

MD simulations capture a diverse range of crucial biomolecular processes that include structural changes and protein folding, alongside forecasting the impacts of mutations on the structure of the protein (Idrees et al., 2017; Hollingsworth and Dror, 2018; Idrees et al., 2022). Therefore, the simulations were conducted on WT-hSOD1, E49K, and R115G mutants (holo dimer forms) to assess the structural dynamic changes. The root-mean-square deviation (RMSD) was applied to quantify the difference between the initial structural conformation and the final positions of the Cα atoms in the protein backbone. The RMSD calculation was carried out to evaluate the changes in the structure and dynamics of the modeled protein (Aier et al., 2016). The backbone RMSD of WT-hSOD1, E49K, and R115G mutants of the proteins were calculated over 300 ns simulations (Figure 4A) and pointed to an unstable behavior at the beginning of simulations. After approximately 150 ns, E49K, and R115G mutants, the structures were floating in a stable state. In contrast, the RMSD value for the WT-SOD1 demonstrated fluctuation for the remainder of the MD trajectory. The average root-mean-square deviation (RMSD) values for WT-SOD1, E49K, and R115G mutants were (0.27 nm), (0.24 nm), and (0.22 nm), respectively. These changes in mutants demonstrated the loss of protein conformational deviation as compared to the WT-SOD1. Our results concerning the RMSD of SOD1 mutants align with previous research on different mutants, which exhibited a relative conformational deviation compared to the WT-SOD1 (Srinivasan and Rajasekaran, 2017; 2019). Besides, hydrogen bonds, one of the most common noncovalent interactions in proteins, play an extremely important role in protein structure and function (Adhav and Saikrishnan, 2023). They were also the first bonds to react to the structural alterations caused by various protein mutations (Alemasov et al., 2017). To determine the potential impact of mutations on the conformational changes in SOD1, the number of hydrogen bonds was computed. The mean number of H-bonds for the holo dimeric WT-SOD1, E49K, and R115G mutations were 204, 202, and 199, respectively (Figure 4B). The number of hydrogen bonds in the mutants is significantly decreased compared to the WT-SOD1, which may influence their structural stability and function, potentially leading to misfolding and aggregation. Previous studies indicate that the removal of hydrogen bonds considerably decreases the stability of SOD1 and promotes aggregation (Tompa et al., 2020; Noorbakhsh Varnosfaderani et al., 2023). Correspondingly, protein stability and the number of intramolecular interactions are both affected by the relative compactness of the protein structure, which is shown by the protein’s radius of gyration (Rg) (Wise-Scira et al., 2013). According to the simulation data presented in Figure 4C, the Rg value computed for the E49K mutant indicated a phase of structural instability, which was observed in the first half of the simulation. After approximately 150 ns, the Rg value exhibited stable behavior for the remainder of the MD trajectory. In comparison, the Rg values for both the WT-SOD1 and mutant R115G displayed initial structural instability, which persisted throughout the entirety of their respective simulations. This analysis also indicated that the average Rg values for the WT-SOD1 (2.07 nm) are relatively similar to those of mutants E49K (2.02 nm) and R115G (2.04 nm). This result suggests that there are slightly increased compactness alterations in the analyzed mutants compared to WT-SOD1. Our findings are in agreement with previous studies (Kalia et al., 2023). In this context, the solvent-accessible surface area (SASA) refers to the exposed regions of protein structures that are accessible to solvent molecules, a key factor in studying protein folding and stability. Amino acid residues in a protein may be categorized as exposed or buried based on their solvent-accessible surface area (SASA) values (Ausaf Ali et al., 2014). The SASA values acquired from all simulations (Figure 4D) showed an unstable behavior at the beginning of all simulations. Following approximately 150 ns, the SASA values exhibited stable behavior until the end of the MD trajectories. This analysis indicated that the average SASA values for the WT-SOD1 (152.78 nm^2^) were similar to those of the mutants E49K (152.27 nm^2^) and R115G (153.21 nm^2^). This finding implies that no significant changes in SASA occurred among the examined mutants compared to WT-SOD1. The findings of our research are in agreement with the previous study, which indicated that no SASA alterations in the analyzed mutants when compared to the WT-SOD1 (Pereira et al., 2021). Next, root-mean-square fluctuation (RMSF) was used to assess the residual flexibility of the WT-SOD1 E49K and R115G mutants (holo-dimer forms). Of note, RMSF, a critical parameter, provides information about the structural flexibility of the Cα atoms in each residue of the corresponding system through MD simulation (Haq et al., 2017). As seen in Figures 5A, B, both monomers displayed asymmetric fluctuation patterns. Specifically, the WT-SOD1 has notable fluctuations in chain A at residues 62–77, which are located in the zinc-binding region of loop IV. Notably, in the E49K and R115G mutants, all atoms contribute almost equally to their relatively stable fluctuations. The average residual RMSF for the WT-SOD1 and mutants in the A and F chains are presented in Table 3. These results suggested that WT-SOD1 is more flexible than the mutants in the holo-dimer form. We find our data to correspond with the results of the previous study, which indicated several mutants have less fluctuation relative to WT-SOD1 (Srinivasan and Rajasekaran, 2019). Secondary structure is a vital component in understanding the conformational stability and functionality of proteins (Kumar et al., 2021). The composition of secondary structures is pivotal in the aggregation process of proteins linked to neurodegenerative diseases (Zaji et al., 2023). Moreover, the transition of the alpha helix into beta-sheets is regarded as a primary issue in the formation of amyloid fibrils, which directly correlates with an increase in aggregation propensity (Dong et al., 2016; Srinivasan and Rajasekaran, 2019). Given this context, the analysis of the secondary structure content for the WT-SOD1, E49K, and R115G mutants was performed using the Dictionary of Secondary Structure in Proteins (DSSP) algorithm to monitor the secondary structural changes throughout the simulation (Table 4). The analysis of the results revealed that the alpha helix arrangement in E49K and R115G mutants was lower than that of WT-SOD1. Conversely, we found that the propensity of beta-sheets was slightly higher in E49K and R115G mutants compared to WT-SOD1. Consequently, we could suggest that E49K and R115G mutants compared to the WT-SOD1 have unequal proportions of different secondary structures, which might be detrimental not only in altering protein stability and function but also in inducing misfolding in SOD1, ultimately leading to protein aggregation. We found that our findings are in harmony with those identified in prior studies (Zaji et al., 2023; Ashkaran et al., 2024). A common multivariate statistical method for minimizing the number of dimensions describing the dominant protein domain motion is Principal Component Analysis (PCA) (Kalia et al., 2023). The projections for the MD trajectories of WT-SOD1, E49K, and R115G mutants into the subspace spanned by PC1 and PC2. As shown in Figures 6A, B, the E49K and R115G mutants occupied a smaller area in conformational space. We also observed changes in cluster shape in the conformational space of both mutants compared to the WT-SOD1. The analysis thus pointed to alternations in the overall essential dynamics of both mutants. Our findings are in agreement with previous studies (Pereira et al., 2021). Cluster analysis of molecular simulation trajectories for molecular systems where the conformation and orientation of the sampled states are important parameters requires a different approach from the standard clustering methods. These systems require cluster analysis methods that discriminate based on molecular orientation and conformation. New clustering methods that account for these parameters are presented and demonstrated by the analysis of trajectories produced in protein simulations for validation (Abramyan et al., 2016). To better visualize the effect of mutation on SOD1 structure, we analyzed the popular conformations obtained using the clustering method with a 0.2 nm cutoff. To further compare the WT-SOD1 with the variant systems, we performed a hierarchical clustering analysis, which allowed us to compare each system with all the others. Different clustering was observed in the wild-type and mutant proteins, with cluster classifications ranging from 5 to 10. The number of clusters for the WT-SOD1, E49K, and R115G mutants was 10, 5, and 6, respectively. The fewer number of clusters may imply a faster aggregation process. On the other hand, the highest conformations for the WT-SOD1, E49K, and R115G mutants was 1916, 2,889, and 2,688, respectively. The most populated conformations are shown as cluster 1 for the WT-SOD1 and mutants in Figure 7.

**TABLE 3:** The average value computed for RMSF of WT-SOD1 and mutants (E49K and R115G).

Protein	RMSF average value (chain A)	RMSF average value (chain F)
	(nm)	
WT-SOD1	0.18	0.15
E49K	0.13	0.13
R115G	0.14	0.14

**TABLE 4:** Percentages of the secondary structure contents of WT-SOD1 and mutants (E49K and R115G).

Protein	Coil	β-Sheet	β-Bridge	Bend	Turn	α-Helix	3_10_-helix
WT-SOD1	27	38	2	16	12	3	2
E49K	27	39	2	18	11	1	2
R115G	26	39	3	19	10	1	2

#### 3.1.5 FEL calculation

**FIGURE 8:**
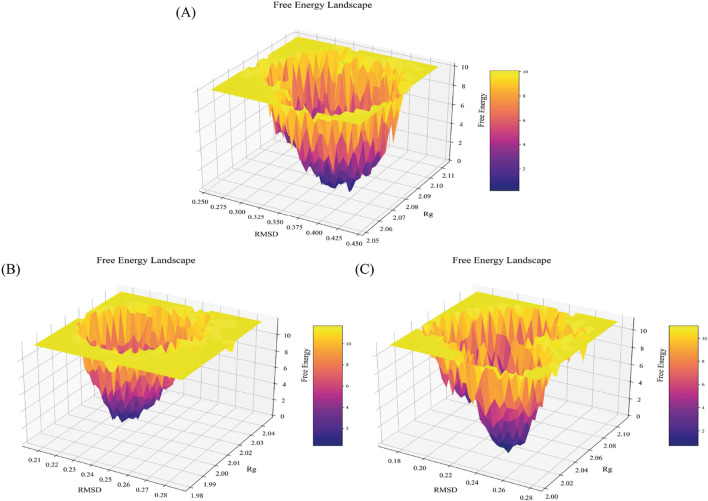
Free energy landscape. FEL for the **(A)** WT-SOD1, **(B)** E49K, and **(C)** R115G mutants. The FEL was constructed with Rg and RMSD as reaction coordinates and energy in Z-axes with values illustrated in the color bar.

Protein aggregation can be well evaluated through the multiple conformational states occupied in the free energy landscape (FEL) (Zheng et al., 2013). Misfolding of protopathic proteins is a key biomolecular trigger for amyloid formation, ultimately leading to amyloid fibrils (Adamcik and Mezzenga, 2018). Accordingly, we separately evaluated the FEL for the WT-SOD1 and mutants. Similarly, a FEL was constructed to enumerate the occurrence of global energy minima constructed to investigate the pathology of the E49K and R115G mutants during the simulation period (Yang et al., 2013). RMSD and Rg were the two coordinates used for FEL analysis to show the compaction and structural stability of the protein during the molecular dynamics period. The Gibbs free energy of the generated FEL of WT-SOD1, E49K, and R115G mutants ranged from 0 to 10 kcal/mol, as shown in Figure 8. The free energy ranges from purple to yellow, challenging the purple region for the minimum global energy composition and the yellow for metastable states. We concluded that the substitution mutation in SOD1 significantly altered the folding pattern, possibly leading to increased RMSD values toward the multiple energy minimum achieved by the mutant conformers. The minimum global energy number for a native protein is usually limited to one. The free energy landscape for the WT-SOD1 structure shows a unique favorable region (Figure 8A) that lies between Rg and RMSD values of ∼2.08 nm and ∼0.38 nm, respectively. The results for the E49K mutant showed a global energy minimum that could occur in more compact conformational states, each of which could cause misfolding and lead to aggregation. The formation of favorable free energy domains (Figure 8B) was 2.02 and ∼0.23 nm for Rg and RMSD, respectively. However, results for the R115G mutant protein indicated multiple global energy minima that could occur in various thermodynamically conceivable conformational states, any of which can lead to aggregation. The effect of substitution mutation on this process led to the formation of numerous favorable free energy basins (Figure 8C), which were ∼2.03 and ∼0.25 nm for Rg and RMSD, respectively. Hence, as the free energy profile showed, the presence of R115 in the wild-type plays a prominent role in the presence of the limited free energy basin along RMSD and Rg, which by replacing Gly led to the change of SOD1 proteins and reaching several global free energy minima. Furthermore, the free energy domain obtained by WT-SOD1 was smaller than the conformational structures of the R115G mutant, indicating a more favorable conformation adopted by the wild-type conformers than the mutant. Altogether, the results support the idea that an increased percentage of multiple conformers with lower free energy in the mutant than the wild-type are directed toward forming predominantly unfolded states. Furthermore, in confirmation of our results indirectly compared to previous studies, it suggests that the aggregated proteins acquire the minimum multiple energy for the conformational structures that indicate the formation of toxic aggregates in mutant SOD1 (Maitham Qassim et al., 2023; Noorbakhsh Varnosfaderani et al., 2023; Abdullah Waheed et al., 2024).

#### 3.1.6 Quantification of SOD1 dimerization using MM/PBSA and per-residue free energy decomposition

**FIGURE 9:**
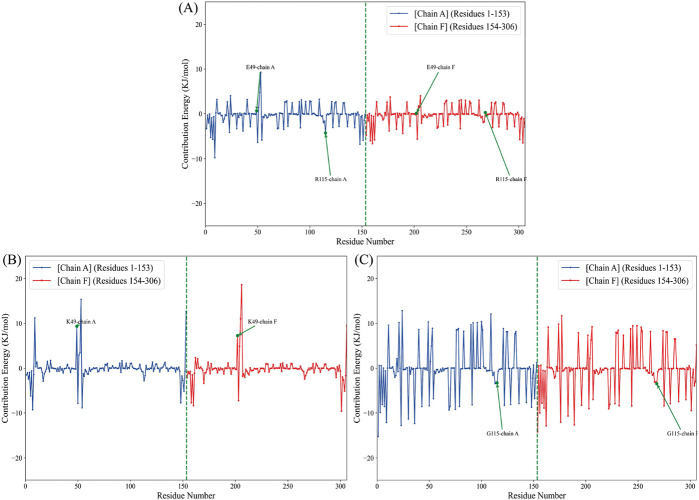
Per-residue decomposition of the binding free energy for the **(A)** WT-SOD1, **(B)** E49K, and **(C)** R115G mutants in chains A (blue line) and F (red line).

The MM-PBSA strategy for binding free energy estimation has become one of the most adopted techniques to measure interaction energies in biomolecular studies (Wang et al., 2019). To quantify SOD1 dimerization in terms of energy, an analysis of MM-PBSA was performed. The results identified the WT-SOD1 system as the most stable form with the highest binding energy, −105.17 kcal/mol. The results further suggested that E49K and R115G mutants compromised the dimerization potential between monomers, leading to reduced binding energies of −32.1 and −79.47 kcal/mol, respectively. The binding energies in the mutants are significantly decreased compared to the WT-SOD1, which may influence their dimer structural stability, potentially leading to aggregation. Our results concerning the MM-PBSA of SOD1 mutants align with previous research on several mutants, which exhibited mutations that compromised the dimerization potential between monomers, leading to reduced binding energies compared to WT-SOD1 (Basith et al., 2022). The technique of per-residue binding free energy decomposition can reveal the contributions of the key residues responsible for the protein-protein interactions at the interface. A total of 1,000 frames extracted from the last 50 ns of the MD trajectories were decomposed by the MM-PBSA method. The analysis of free energy decomposition was performed on the WT-SOD1 and mutants. Free energy decomposition analysis helps to find the contribution of a single residue by summing its interactions over the entire residues. The results of this study showed that in the wild-type state (Figure 9A), the binding free energy for residues E49 and R115 in chain-A was 0.64 and −4.27 kJ/mol, respectively. For chain-F, the binding energy values for residues E49 and R115 were 0.06 and 0.26 kJ/mol, respectively. The E49K mutation (Figure 9B) changed the binding free energy value, which was 9.31 and 7.25 kJ/mol in the A and F chains, respectively. On the other hand, the binding free energy values for the R115G mutation (Figure 9C) were −3.26 and −3.43 kJ/mol in chains A and F, respectively. The analysis thus pointed to alternations in the per-residue free energy decomposition of both mutants compared to the WT-SOD1, which may influence their dimer structural stability, potentially leading to aggregation.

### 3.2 Experimental section

#### 3.2.1 Enzymatic activity assay

Almost all mutations in SOD1-amyotrophic lateral sclerosis result in a significant decrease in SOD1 enzyme activity, according to published data from individuals with this disease (Saccon et al., 2013). The active site of the SOD1 protein contains a Cu^2+^ ion coordinated with four histidine residues: His 46, His 48, His 120, and His 63 (Keerthana and Kolandaivel, 2015). The enzymatic activity of WT-hSOD1, E49K, and R115G mutants was 7,031, 5,536, and 4305 U/mg, respectively. The results of our study revealed reduced enzymatic activity for E49K and R115G compared to holo WT-hSOD1. At position 49, glutamic acid has a negative charge and is vital for setting up the electrostatic environment that His48 needs to coordinate the copper ion. This contact is essential for the enzyme’s performance. The E49K mutation substitutes essential glutamic acid with positively charged lysine. This change may substantially disturb the delicate balance necessary for the correct coordination of the his48 with the copper ion. On the other hand, the R115G mutation diminishes the van der Waals interaction with His48. The lack of this interaction may further affect the structural integrity and activity of the enzyme, especially considering the significance of copper coordination at the His48 location. Initially, researchers believed that ALS-causing SOD1 mutations altered the protein’s structure, resulting in an unstable or misfolded enzyme that lowered dismutase activity and heightened oxidative stress. However, different FALS-linked SOD1 mutations had different amounts of dismutase activity. This suggests that mutant SOD1’s toxicity could not be explained by a simple loss of function. Additionally, there are no FALS patients known to have mutations resulting in complete loss of SOD1 (Barber et al., 2006). Our results correspond with previous investigations indicating that the specific activity of several SOD1 mutants is decreased in comparison to the WT-SOD1 (Mavadat et al., 2023b; Ashkaran et al., 2024).

#### 3.2.2 Fluorescence and FTIR spectroscopy analysis

**FIGURE 10:**
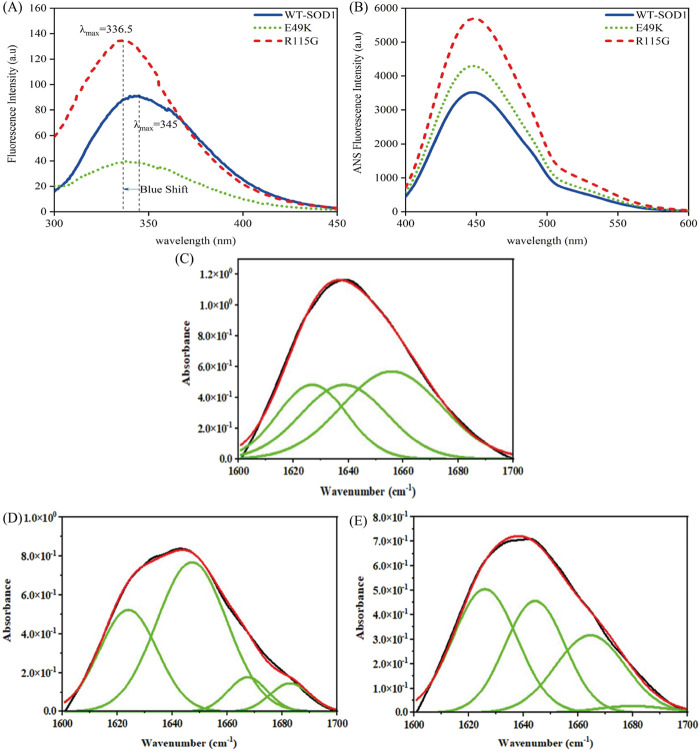
Structural characteristics of WT-hSOD1 and mutants of SOD1 (E49K, R115G) by **(A)** intrinsic fluorescence and **(B)** extrinsic fluorescence emission in phosphate buffer. Results of a Fourier self-deconvolution (FSD) analysis on the amide I (1,600–1,700 cm^−1^ region) for **(C)** WT-SOD1, **(D)** E49K, and **(E)** R115G mutants. The assignment of different peaks to different secondary structure elements is shown. Protein concentration was 25 μM in the presence of amyloidogenic conditions. For further details, please refer to the *Materials and Methods* section.

Among the aromatic amino acids (Trp, Tyr, and Phe), intrinsic Trp fluorescence is the most often used method to track local structural and dynamic changes in proteins (Raghuraman et al., 2019). WT-hSOD1 only possesses one Trp at residue 32 (W32), which is highly solvent-exposed (Grad et al., 2011). Figure 10A, A shows the intrinsic fluorescence of the E49K mutant is lower than that of WT-hSOD1, indicating that the environment of the single solvent-exposed Trp residue 32 is not buried. In other words, the polarity of the Trp residue microenvironment increased and was more exposed to the solvent. The R115G mutant fluorescence spectrum shows an increase in fluorescence intensity with a change of λmax in the emission spectra and a blue shift compared to the wild-type, showing that the alterations in the circumambient environment, the tryptophan (W32), toward a more hydrophobic environment. The ability to measure the hydrophobicity of a protein may be advantageous in comprehending and forecasting the implications of alterations to the sequence of its structure. The increased ANS fluorescence signified that ANS bound to the exposed contiguous hydrophobic regions (Cardamone and Puri, 1992; Idrees et al., 2016). The hydrophobic inner core of the monomer SOD1 contains roughly half of the protein backbone (Wright et al., 2019), and protein dimerization provides approximately 640 Å of hydrophobic surface area at the dimer interface through a complex network of hydrogen bonds and hydrophobic interactions, resulting in a compact mature homodimer with minimal solvent accessibility. Accordingly, holo-SOD1 is one of the most stable proteins known (Trist et al., 2021). To evaluate the impact of mutations on changes in SOD1’s hydrophobicity, ANS fluorescence was utilized. As shown in Figure 10B, the ANS fluorescence of E49K and R115G indicates a significant increase compared to the WT-SOD1, indicating the role of mutations in conformational changes and increased amounts of exposed hydrophobic surfaces. Our findings are in agreement with previous studies (Namadyan et al., 2023). FTIR, which is known as one of the accurate techniques for reflecting the secondary structure of proteins, was used to study misfolding and aggregate formation (Miller et al., 2013). Accordingly, the formation of β-sheet structures in WT-SOD1 and the mutants (under destabilizing conditions) was confirmed by FTIR spectroscopy (Figures 10C–E). The FTIR spectra were also employed to distinguish between types of β-sheet structures and the amyloidogenic conformers (amide I bands) were then determined. According to Table 5, the peak in 1,610–1,630 cm^−1^, indicating the formation of fibrils and β-oligomers with the intermolecular β-sheet structure due to protein aggregation, was reported for WT-SOD1 and mutants. The native β-sheets could produce an absorption peak in the 1,623–1,641 cm^−1^ range. Examining the amide (I) bands, a parallel arrangement could be thus marked in the anti-parallel arrangement of the β-strands in the protein aggregates. In this line, the parallel β-sheets could display only an elevated component at 1,623–1,641 cm^−1^ (Hoffner et al., 2014). The disordered conformation (random coil) is usually associated with the IR band between 1,640 and 1,648 cm^−1^. A distinctive feature of a random coil is that it is non-repetitive. Such an assignment is supported by the position of a prominent band in the spectrum of apparently disordered proteins (Byler and Susi, 1986). A component centered at approximately 1,650–1,660 cm^−1^ is assigned to the α-helix, which is consistent with theoretical calculations and the observation of bands in the spectrum of α-helix proteins (Sadat and Joye, 2020). α-Helix structure was observed for WT-SOD1 at peak position 1,655, cm^−1^. On the other hand, α-helical structure (3_10_-helix) was observed for the E49K and R115G mutants at peak positions of 1,666 and 1,664 cm^−1^, respectively. Bands near 1,663 ± 3 cm^−1^ are assigned to 3_10_ helices, although this structure is rarely found in proteins (Dong et al., 1990). The 3_10_-helix structure is classified as a subset of the α-helix and appears in proteins when the α-helix is distorted due to the presence of (mutation) an undesirable residue (Peters, 2005). The assignment of bands around 1,670–1,699 cm^−1^ to β-turns has been suggested (Krimm and Bandekar, 1986). Hence, the β-turn structure was observed for the E49K and R115G mutants in the peak region of 1,682 and 1,679 cm^−1^, respectively. Therefore, the FTIR spectroscopy results showed that the mutants have different changes in the natural β-sheet structure of the protein compared to the wild-type. Therefore, the changes in the secondary structure of the SOD1 variants indicate a structural rearrangement of SOD1, potentially leading to aggregation. The results are consistent with what has been previously reported (Ashkaran et al., 2024; Farrokhzad et al., 2024).

**TABLE 5:** Assignment of amide I band components and percentages of secondary structure contents in WT-SOD1 and mutants (E49K and R115G).

Protein	Corresponding secondary structure	Observed negative peaks (cm^−1^)	Secondary structure (%)
WT-SOD1	β-Sheet (aggregated)	**1,626**	25.22
	β-Sheet	1,638	31.56
	α-Helix	1,655	43.22
E49K	β-Sheet (aggregated)	**1,623**	31
	Random coil	1,647	55.81
	α-Helix (3_10_ helix)	1,666	7.19
	β-Turn	1,682	6
R115G	β-Sheet (aggregated)	**1,625**	38.39
	Random coil	1,644	32.9
	α-Helix (3_10_ helix)	1,664	26.54
	β-Turn	1,679	2.17

Note: Bolded values indicate β-sheet (due to aggregation) or cross-β structure.

#### 3.2.3 Fluorescence spectroscopy analysis under destabilizing circumstances

**FIGURE 11:**
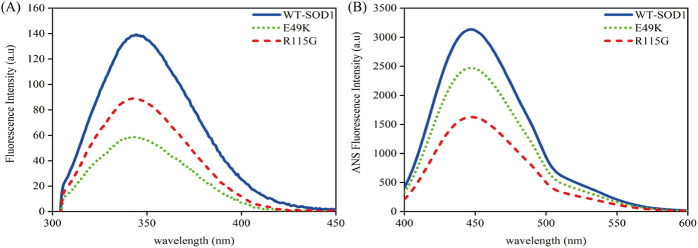
Structural characteristics of WT-hSOD1 and mutants of SOD1 (E49K, R115G) by **(A)** intrinsic fluorescence and **(B)** extrinsic fluorescence emission in the presence of amyloidogenic conditions. For further details, please refer to the *Materials and Methods* section.

To obtain a deeper understanding about the relationship between aggregate formation and changes in the Trp environment, we looked at the intrinsic fluorescence of pathological aggregates formed from wild-type and mutants under destabilizing circumstances. Obtained data indicated that fluorescence intensity in both mutants during aggregation compared to wild-type shows that Trp-32 is not buried. In other words, the polarity of the Trp residue microenvironment increased and was more exposed to the solvent, as seen in Figure 11A. To evaluate the impact of mutations on changes in SOD1’s hydrophobicity, as well as to observe the exposure of hydrophobic pockets in β-sheet structures during aggregation formation, ANS fluorescence was utilized. Several factors, such as formulation conditions, protein sequences, and structure, influence protein aggregation. The classical hydrophobic effect drives the interaction of exposed hydrophobic pockets on the protein surface, leading to the formation of protein aggregates (Tosstorff et al., 2019). According to Figure 11B, ANS fluorescence emission of mutants under amyloid induction conditions showed lower emission intensity than WT-SOD1. The decrease in ANS fluorescence intensity is probably due to the coverage of large parts of the exposed hydrophobic surfaces, which results from the interaction of hydrophobic segments between protein molecules during the amyloid process. These results are aligned with observations noted in earlier studies (Abbasabadi et al., 2013; Salehi et al., 2015; Baziyar et al., 2022; Mavadat et al., 2023b).

#### 3.2.4 ThT fluorescence assay

**FIGURE 12:**
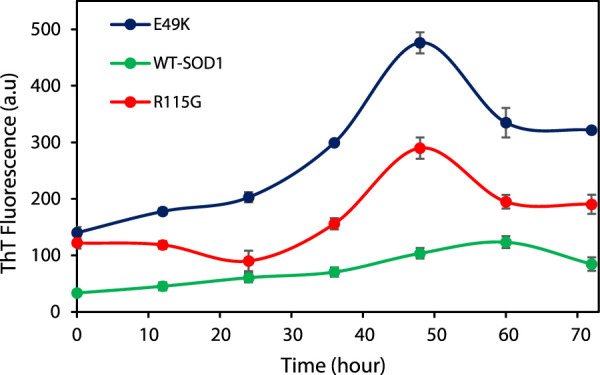
Kinetics of WT-SOD1, E49K and R115G mutants’ amyloid aggregation Formation using ThT fluorescence incubated in the presence of 30 μM protein in 20 mM phosphate buffer, ThT concentration 20 μM and under induction conditions (DTT 50 mM, Tris-HCl 50 mM, KSCN 0.16 M) pH 7.4 at 37°C with and without agitation at 190 rpm. Data are from two independent replicates.

The end-point level of ThT fluorescence intensity has been on mature fibrils, as generally reported. A growing number of structural models for amyloid fibrils thus strongly suggest that ThT binds to the long axis of fibril by aligning parallel, intercalating to the repeating side-chain interactions running across β-strands within the β-sheet layer (Wu et al., 2009). In this study, WT-SOD1 and mutants were incubated under inducing conditions for time intervals of 0–72 h with and without agitation. Once NaCl was substituted for KSCN, no amyloid was formed. This implied that the effect of KSCN was related to its chaotropic properties but not its impact on ionic strength. The representative fluorescence time courses for the WT-SOD1 and mutants are illustrated in Figure 12 and a complete set of the kinetic parameters are then given in Table 6. The results showed that the WT-SOD1 and mutants formed amyloid aggregates when there were high concentrations of DTT. Furthermore, experiments showed that SOD1 fibrillation preceded the growth in ThT fluorescence, which was different in mutant and WT-SOD1. This delay was different in mutants and WT-SOD1. Comparison of the lag phases showed that the mutants tended to have shorter lag phases and faster fibrillation than holoWT-SOD1. This step increased thioflavin T fluorescence to different levels for WT-SOD1 and mutants. However, this change in lag was shown to be compatible with other reported results that have been studied under different settings (Abbasabadi et al., 2013; Salehi et al., 2015). In particular, FALS-causing mutations wouldn't always reduce the latency and/or increase the aggregation propensity of SOD1. In addition, the kinetics of amyloid fibril aggregation is delayed, with an initial increase in ThT fluorescence intensity, which was more pronounced for mutants than for WT-SOD1, followed by a decrease in ThT intensity. This may be due to the formation of mat-like aggregates of fibrils, resulting in a number of modified or unavailable binding sites for ThT. This difference in the kinetics of aggregate formation in the mutant forms may be due to the amino acid substitution. Finally, when the ThT fluorescence intensity reaches its maximum, the plateau phase begins. However, this stage does not last long because amyloid fibers tend to form web-like network structures. The findings of our study may support the idea that SOD1 aggregation occurs through a pathway including weakening of the dimer interface, dimer dissociation, reducing the binding/affinity of metal ions from monomers, and aggregation of monomers (Tiwari and Hayward, 2003; Hough et al., 2004). Furthermore, the results show that SOD1 forms amyloid aggregates under different conditions including agitation, ionic strength, temperature and physiological pH. We were able to demonstrate the potential of WT-SOD1 and mutants to form amyloid structures under conditions inducing amyloid aggregation. These results are consistent with previous studies showing the importance of fibrillation or oligomerization of SOD1 in the pathogenesis of ALS (Oztug Durer et al., 2009; Abbasabadi et al., 2013; Salehi et al., 2015; Baziyar et al., 2022).

**TABLE 6:** Kinetic parameters of amyloid aggregation formation from WT-SOD1 and mutants in 50 mM Tris-HCl, 0.16 M KSCN, and 50 mM DTT at pH 7.4 with agitation at 190 rpm at 37°C.

Protein	Lag time (h)	K_app_ (h^−1^)
WT-SOD1	32 ± 3	0.111 ± 0.025
E49K	26 ± 2	0.181 ± 0.013
R115G	27 ± 4	0.166 ± 0.028

## 4 Conclusion

The present study focused on investigating the potential effect of the two-point mutations at the surface (namely, E49K and R115G), which are located in metal-binding loop IV and Greek key loop VI, respectively, on the structure and dynamics of the SOD1 molecule using computational predictions, molecular dynamics simulations, and experimental studies. The *in silico* analysis utilizing various algorithms revealed a high rate of deleterious and destabilizing predictions for these mutants, suggesting their harmful effects. The findings obtained for the molecular modeling showed that E49K and R115G mutants significantly changed the distribution of the net negative charge and interaction of adjacent amino acids, respectively, compared to the WT-SOD1, which could reduce the stability of the SOD1 dimer, promoting aberrant interactions and misfolding. The MD analyses of variants E49K and R115G pointed to structural destabilization by affecting the increased content of β-sheet structures and loss of hydrogen bonds that could lead to protein misfolding. Furthermore, the results of the experimental section demonstrated that hSOD1 mutations change the protein structure and conformation, which leads to exposing hydrophobic pockets that would typically be buried within the core of the protein structure which could affect the stability and ultimately promote protein aggregation. In addition, experimental analysis illustrated that conformational changes in mutants could affect the enzyme activity compared to the WT-SOD1. In summary, our study highlights key differences in the structure and dynamics of investigated SOD1 variants and WT-hSOD1. It sheds light on the behavior of SOD1 variants at an atomic and molecular level, suggesting that mutations in the metal-binding loop IV and Greek key loop VI lead to induced significant structural and conformational changes that could affect the structure and stability of the SOD1 molecule, which ultimately results in enhanced formation of toxic aggregates and the pathology of ALS. The structural insights obtained from this study will assist future experimental and computational research focused on targeted therapies. This study elucidated the processes of protein instability and aggregation, establishing a basis for the investigation of therapeutic agents that may stabilize SOD1 mutants or prevent and/or decrease protein aggregate formation related to ALS disease.

## Data Availability

The original contributions presented in the study are included in the article/supplementary material, further inquiries can be directed to the corresponding author.
